# Can an initial end-tidal CO_2_ <1.33 kPa predict lack of return of spontaneous circulation during pre-hospital cardiac arrest?

**DOI:** 10.1186/1757-7241-22-S1-O5

**Published:** 2014-07-07

**Authors:** Leif Rognås, Troels Martin Hansen, Hans Kirkegaard, Else Tønnesen

**Affiliations:** 1 Department of research and development, Norwegian Air Ambulance Foundation, Drøbak, Norway; 2 Pre-hospital Critical Care Team, Aarhus University Hospital, Aarhus, Denmark; 3 Centre for Emergency Medicine Research, Aarhus University Hospital, Aarhus, Denmark; 4 Department of Anaesthesiology, Aarhus University Hospital Aarhus, Denmark

## Background

Previous results have indicated that an initial end-tidal (ET) CO_2_ < 1.33 may be used as a cut-off value for when return of spontaneous circulation (ROSC) can be achieved during pre-hospital cardiac arrest [1].

We aimed at validating these results in our anaesthesiologist-staffed pre-hospital critical care system.

## Materials and methods

We prospectively registered data [2] according to the Utstein-style template for reporting data from pre-hospital advanced airway management [3] from February 1^st^ 2011 to October 31^st^ 2012. Included were consecutive patients at all ages with pre-hospital cardiac arrest treated by eight anaesthesiologist-staffed pre-hospital critical care teams in the Central Denmark Region.

## Results

We registered data from 595 cardiac arrest patients; in 58.9 % (n=350) of these cases the pre-hospital critical care teams performed pre-hospital endotracheal intubation.

An initial end-tidal CO_2_ measurement following pre-hospital endotracheal intubation were available in 270 cases.

We identified 22 patients, who had an initial ETCO_2_ below 1.33 kPa.

Four of these patients achieved return of spontaneous circulation. All four patients were admitted to hospital, three with stable circulation and one with ongoing automated CPR due to recurrent cardiac arrest.

## Conclusion

Our results indicates that an initial ETCO_2_ below 1.33 kPa during pre-hospital cardiac arrest should not be used as a cut-off value for the achievability of return of spontaneous circulation.
